# Association of glucose uptake of visceral fat and acute myocardial infarction: a pilot ^18^F-FDG PET/CT study

**DOI:** 10.1186/s12933-020-01115-3

**Published:** 2020-09-24

**Authors:** Kisoo Pahk, Eung Ju Kim, Chanmin Joung, Hong Seog Seo, Sungeun Kim

**Affiliations:** 1 Department of Nuclear Medicine, Korea University Anam Hospital, 73, Inchon-ro, Seongbuk-gu, Seoul, 02841 Republic of Korea; 2 Department of Cardiovascular Center, Korea University Guro Hospital, 148, Gurodong-ro, Guro-gu, Seoul, 08308 Republic of Korea; 3 Institute for Inflammation Control, Korea University, Seoul, 02841 Republic of Korea

**Keywords:** Coronary artery disease, Visceral fat, Inflammation, Atherosclerosis, Positron-emission tomography

## Abstract

**Background:**

Inflamed visceral adipose tissue (VAT) facilitates chronic inflammation in atherosclerotic lesions thereby leading to increased risk of coronary artery disease (CAD). In this study, we evaluated the glucose uptake of VAT and the carotid artery with ^18^F-fluorodeoxyglucose positron emission tomography (^18^F-FDG PET/CT) and their association with CAD, including acute myocardial infarction (AMI).

**Methods:**

A total of 90 participants were enrolled (32 with AMI, 33 with chronic stable angina; CSA, and 25 control participants) and undertook ^18^F-FDG PET/CT. VAT glucose uptake was measured by using maximum standardized uptake value (SUVmax) of VAT region. The target-to-background ratio (TBR) of carotid artery was defined as the SUVmax of carotid artery divided by the SUVmax of jugular vein. The SUVmax of spleen, bone-marrow (BM), and high-sensitivity C-reactive protein (hsCRP) were used for the assessment of systemic inflammatory activity.

**Results:**

VAT SUVmax was highest in participants with AMI, intermediate in participants with CSA, and lowest in control participants. Carotid artery TBR and systemic inflammatory surrogate markers including spleen SUVmax, BM SUVmax, and hsCRP were also higher in the AMI group than in the CSA or control group. Furthermore, VAT SUVmax showed significant positive correlation with carotid artery TBR, spleen SUVmax, BM SUVmax, and hsCRP. In multivariate linear regression and logistic regression analyses, VAT SUVmax was independently associated with carotid artery TBR and AMI.

**Conclusions:**

Glucose uptake of VAT assessed by ^18^F-FDG PET/CT was associated with the severity of CAD and synchronized with the carotid artery inflammation in participants with CAD.

## Background

Cardiovascular diseases (CVD) are the leading cause of mortality in worldwide and approximately 40% of these deaths are attributed to coronary artery disease (CAD) [1, 2]. One of the main underlying pathological processes leading to CAD is atherosclerosis which can further develop into plaque erosion or rupture that eventually manifest as angina and/or acute myocardial infarction (AMI) [3]. Furthermore, accumulating evidences suggest that dysfunctional visceral adipose tissue (VAT) is the key player underlying the risk of CAD development through the promotion of chronic inflammation in arterial lesions with atherosclerosis [4, 5].

Obesity, especially visceral obesity, has been well known to transform healthy VAT into dysfunctional and inflamed VAT [4–6]. Dysfunctional VAT as an endocrine organ is metabolically activated and produces pro-inflammatory cytokines, such as tumor necrosis factor-alpha (TNF- α), interleukin-6 (IL-6), and monocyte chemotactic protein-1 (MCP-1), thereby accelerating infiltration of inflammatory cells, mainly classically activated (M1) macrophages into VAT, which further lead to exacerbation of VAT inflammation [4–6]. Inflamed VAT promotes increasing circulating pro-inflammatory cytokines thereby increasing remote atherosclerotic plaque inflammation such as carotid artery inflammation which leads to development and rupture of atherosclerotic plaques and eventually contributes to an increased risk of CAD and aggravates the severity of CAD [4–6].

^18^F-fluorodeoxyglucose positron emission tomography (^18^F-FDG PET/CT) is a well-known non-invasive imaging modality for measurement of inflammatory activity, especially M1 macrophage activity [7]. This concept has been well established by the study with atherosclerotic vulnerable plaques using ^18^F-FDG PET/CT imaging [7–9]. Furthermore, increased glucose uptake of VAT evaluated by ^18^F-FDG PET/CT has been found to be associated with tumor aggressiveness, for which upregulated VAT inflammation is a known risk factor [10–12]. Thus, ^18^F-FDG PET/CT can be used as a surrogate marker for evaluation of VAT inflammation.

Recently, we found that the VAT glucose uptake assessed by ^18^F-FDG PET/CT is increased proportionally to the number of metabolic syndrome components [13] which are the powerful predictor of CVD [14]. On the basis of these findings, we hypothesized that VAT glucose uptake could also be related with the severity of CAD.

In this study, we aimed to investigate whether the VAT glucose uptake evaluated by ^18^F-FDG PET/CT is related to the severity of CAD and carotid artery inflammation in participants with CAD, including AMI.

## Methods

### Study participants

Participants with diagnosed CAD including AMI or chronic stable angina (CSA) from June 2008 to March 2009 were enrolled in this study. AMI was defined as detection of typical change of biochemical markers reflecting myocardial necrosis together with ≥ 1 of the following: symptoms of ischemia, electrocardiographic changes indicative of new ischemia, development of pathologic Q waves, new regional wall motion abnormality or imaging evidence of new loss of viable myocardium [15]. CSA was defined as the presence of symptoms of stable angina for ≥ 6 months along with ≥ 50% luminal narrowing in at least one major coronal artery on angiographic evaluation [16]. Consecutive age-matched participants who underwent ^18^F-FDG PET/CT for routine health check-up from June 2008 to March 2009 at the Health Promotion Center of Korea University Guro Hospital were enrolled as control group. Control participants with a history of CVD (myocardial infarction, unstable angina, stroke, and/or cardiovascular revascularization), uncontrolled diabetes mellitus (glycated hemoglobin > 9%), greater than stage 1 hypertension (resting blood pressure ≥ 160/100 mmHg), cancers, severe renal or hepatic diseases, history of an inflammatory condition, and/or treated with any inflammation-modulating medications that could affect systemic inflammatory condition within 6 months of present study were excluded. Finally, a total of 90 participants were included and underwent ^18^F-FDG PET/CT. This study conformed to the guidelines of the Declaration of Helsinki and the Institutional Review Board of Korea University Guro Hospital (Approval No. KUGH06114) approved the study design, and all participants provided written informed consent.

### Anthropometric and laboratory measurements

Body mass index (BMI) was calculated as weight/height squared (kg/m^2^), and waist circumference (WC) was measured at the level of umbilicus in the sitting position. All blood samples were obtained after 12-hour overnight fasting. The levels of serum triglyceride, high-density lipoprotein cholesterol, and glycated hemoglobin were measured using a chemistry analyzer (Hitachi 747, Hitachi, Tokyo, Japan), and the low-density lipoprotein cholesterol concentration was calculated using the Friedewald formula [17]. The high-sensitivity C-reactive protein (hsCRP) levels were determined by using a chemiluminescence immunoassay (Beckman Coulter, Brea, CA, USA). Cardiac troponin-T and creatine kinase-MB fraction were measured by using an Elecsys 2010 analyzer (Roche Diagnostics, Indianapolis, IN, USA). According to the instructions of manufacturer, the concentration of troponin-T > 0.1 ng/mL and creatine kinase-MB > 6.73 ng/mL in men or > 3.77 ng/mL in women were determined as cut-off values for diagnosis of AMI.

## ^18^F-FDG PET/CT protocol

All participants were fasted for at least 6 h before undergoing ^18^F-FDG PET/CT to maintain a blood glucose level of < 180 mg/dL. Participants with AMI were all successfully treated with percutaneous coronary intervention and took ^18^F-FDG PET/CT within 10 days of AMI onset when they were clinically stable. Participants with CSA and control group took ^18^F-FDG PET/CT on the scheduled date. Same ^18^F-FDG PET/CT protocol was applied to all three groups. In participants with diabetes mellitus, we tried to achieve normal glycemic values prior to take ^18^F-FDG PET/CT. First, the participants with diabetes mellitus controlled by oral medication were scheduled to perform ^18^F-FDG PET/CT in the late morning with continue to take oral medication to control their glucose level. Second, the participants with diabetes mellitus controlled by insulin were scheduled to perform ^18^F-FDG PET/CT in the early morning with a use of intermediate-acting insulin evening before to minimize the effect of injected insulin on image quality. Then they should eat a normal breakfast after taking ^18^F-FDG PET/CT.

The scan was initiated 60 min after injection of 5.29 MBq/kg ^18^F-FDG using a dedicated PET/CT scanner (GEMINI TF, Philips Medical Systems, Cleveland, OH, USA). The CT scan was performed from head to thigh with a 16-slice helical CT (4 mm thickness; 120 kVp; 50 mA) followed by PET scan for attenuation correction. The images were reconstructed iteratively by a three-dimensional row-action maximum likelihood algorithm.

### Image analysis

Images were analyzed by experienced nuclear medicine physician using commercially available workstation (Extended Brilliance Workspace version 3.5, Philips Healthcare, Eindhoven, Netherlands). For the measurement of carotid artery inflammation, regions of interest (ROIs) were placed on the right carotid artery and the right jugular vein. Standardized uptake value (SUV) was calculated as follows:

$$ SUV \, = \, Tracer \, activity \, \left( {ROI} \right) \, \left( {MBq/mL} \right)/Injected \, dose \, \left( {MBq} \right)/Total \, body \, weight \, \left( g \right) $$

Subsequently, the arterial target-to-background ratio (TBR) was defined as averaged highest carotid artery SUV divided by averaged highest jugular vein SUV over all axial slices [16]. For the measurement of VAT area, VAT area was identified on CT images at the level of L4-L5 based on the predefined Hounsfield units (ranging from − 70 to − 110), as previously described [10–13]. ROI was manually drawn along the border of intra-abdominal fat. For the assessment of VAT glucose uptake, ROIs were placed on the targeted region and the maximum standardized uptake (SUVmax) was acquired. A total of 10 ROIs were located on VAT area and manually adjusted to exclude the overspill ^18^F-FDG uptake in the muscle vessel, and/or intestine, as previously described [10–13]. VAT SUVmax was defined as the averaged SUVmax from those 10 ROIs. For the measurement of subcutaneous adipose tissue (SAT) area, SAT area was identified on CT images at the level of L4-L5 based on the predefined Hounsfield units (ranging from − 70 to − 110) and ROI was placed along the subcutaneous fat. For the assessment of SAT glucose uptake, a total of 10 ROIs were also placed on the anterior abdominal wall or buttock area and averaged SAT SUV from those ROIs were defined as SAT SUVmax. As shown in Table 1, the results of intra-class correlation coefficient (ICC) showed good reproducibility for measurement of adipose tissue glucose uptake between inter- and intra-observer.

**Table 1:** Intra-class correlation coefficient (ICC) analysis for measurement of SUVmax in adipose tissue between inter- and intra-observer

	Inter-observer reliability		Intra-observer reliability	
	ICC	95% CI	ICC	95% CI
VAT SUVmax	0.959	0.937–0.973	0.961	0.941–0.974
SAT SUVmax	0.882	0.82–0.922	0.855	0.78–0.905

*VAT* visceral adipose tissue, *SAT* subcutaneous adipose tissue, *SUVmax* maximum standardized uptake value, *95% CI* 95% confidence interval

Increased glucose uptakes of spleen and bone marrow (BM) assessed by ^18^F-FDG PET/CT can reflect the increased myeloid activity which is accompanied by systemic inflammation thereby being useful as surrogate markers for systemic inflammation [16, 18]. For the assessment of glucose uptake of spleen and bone marrow (BM), targeted ROIs were located on the spleen and L3 to L5 vertebrae. Averaged SUVmax from all axial slices were defined as spleen SUVmax and BM SUVmax, respectively [13, 16].

### Statistical analysis

All data were presented as mean ± standard deviation. Distribution of normality was tested with the Shapiro–Wilk test. Student’s *t* test or Mann–Whitney *U* test was used for comparison of two groups. The Pearson Chi squared (χ^2^) test or Fisher’s exact test, and one-way analysis of variance (ANOVA) with post hoc Tukey test or Kruskal–Wallis test with post hoc Dunn’s test were used for comparison of multiple groups. Spearman’s correlation coefficient, receiver-operating characteristic (ROC) curve analysis, multiple linear regression analysis, and multiple logistic regression analysis were also performed. Data were analyzed using SPSS version 17.0 (SPSS Inc., Chicago, IL, USA) and MedCalc version 18.5 (MedCalc, Mariakerke, Belgium). The statistical power was set at 0.8 and a *p* value of < 0.05 was considered statistically significant.

## Results

### Clinical characteristics

Of the 90 participants, 32 were in the AMI group, 33 were in the CSA group, and 25 were in the control group. Compared with the control group, known traditional CVD risk factors such as diabetes mellitus, hypertension, dyslipidemia, and smoking habit, were more prevalent in CAD group. The prevalence of those CVD risk factors was not significantly different between the AMI and the CSA groups. The baseline characteristics of all participants are presented in Table 2.

**Table 2:** Baseline characteristics of participants

	Control, n = 25	CSA, n = 33	AMI, n = 32	*p*
Age, y	57.1 ± 7.8	61.2 ± 11.5	57 ± 11.6	0.206
Men, n (%)	6 (24)	24 (72.7)*	21 (65.6)^†^	< 0.001
BMI, kg/m^2^	23.5 ± 2.9	26 ± 4*	24.6 ± 2.6	0.021
WC, cm	80.9 ± 7.5	92.3 ± 11.4*	83.4 ± 16.3^‡^	< 0.001
Hypertension, n (%)	1 (4)	19 (57.6)*	15 (46.9)^†^	< 0.001
DM, n (%)	2 (8)	13 (39.4)*	13 (40.6)^†^	0.021
Dyslipidemia, n (%)	2 (8)	16 (48.5)*	19 (59.4)^†^	< 0.001
Current smokers, n (%)	2 (8)	13 (39.4)*	13 (40.6)^†^	0.021
Statin use, n (%)	0	11 (33.3)	9 (28.1)	0.649
Hypertension medication, n (%)	0	17 (51.5)	6 (18.8)^‡^	0.006
DM medication, n (%)	0	13 (39.4)	10 (31.3)	0.492
Total choleaterol, mg/dL	189 ± 25.3	156.4 ± 35.2*	186.9 ± 43.6^‡^	0.001
Triglycerides, mg/dL	86.7 ± 44.3	160.4 ± 99.8*	136.6 ± 142.1^†‡^	< 0.001
HDL cholesterol, mg/dl	59.4 ± 15.7	48.7 ± 15.2*	45 ± 11.8^†^	0.001
LDL cholesterol, mg/dL	115.3 ± 24.1	91.9 ± 30.1*	124.3 ± 41.7^‡^	< 0.001
HbA1c, %	5.7 ± 0.4	7 ± 1.6*	6.9 ± 2.1^†^	< 0.001
WBC, × 10^3^/μL	5 ± 1.3	6.5 ± 1.2*	10.9 ± 3.3^†‡^	< 0.001
hsCRP, mg/L	0.6 ± 0.6	1.5 ± 1.6*	3.5 ± 3.1^†‡^	< 0.001
VAT area, cm^2^	147 ± 57.1	261.3 ± 110.6*	209.1 ± 80.1^†^	< 0.001
SAT area, cm^2^	335.4 ± 95	347.8 ± 168.4	294.2 ± 83.1	0.294
peak CK-MB, ng/mL	…	…	145.6 ± 127.3	…
peak troponin-T, ng/mL	…	…	3.7 ± 4.6	…
Metabolic parameters				
Carotid artery TBR	1.2 ± 0.1	1.4 ± 0.4*	2.1 ± 0.4^†‡^	< 0.001
Spleen SUVmax	1.5 ± 0.3	2 ± 0.3*	2.6 ± 0.4^†‡^	< 0.001
BM SUVmax	0.8 ± 0.4	1.2 ± 0.6*	1.7 ± 0.2^†‡^	< 0.001

All data were presented as mean ± standard deviation or n (%). *P*-values were determined using ANOVA with post hoc Tukey test or Kruskal–Wallis test with post hoc Dunn’s test for continuous variables and Pearson Chi squared (χ^2^) test or Fisher exact test for categorical variables. *p < 0.05, Control vs. CSA, ^†^*p* < 0.05, Control vs. AMI, ^‡^*p* < 0.05, CSA vs. AMI. *CSA* chronic stable angina, *AMI* acute myocardial infarction, *BMI* body mass index, *WC* waist circumference, *DM* diabetes mellitus, *HDL* high-density lipoprotein, *LDL* low-density lipoprotein, *HbA1c* hemoglobin A1c, *WBC* white blood cell, *hsCRP* high-sensitivity C-reactive protein, *VAT* visceral adipose tissue; *SAT* subcutaneous adipose tissue, *CK*-*MB* creatine kinase-MB, *TBR* target-to-background ratio, *SUVmax* maximum standardized uptake value, *BM* bone marrow

### VAT glucose uptake is increased in CAD

**Fig. 1:**
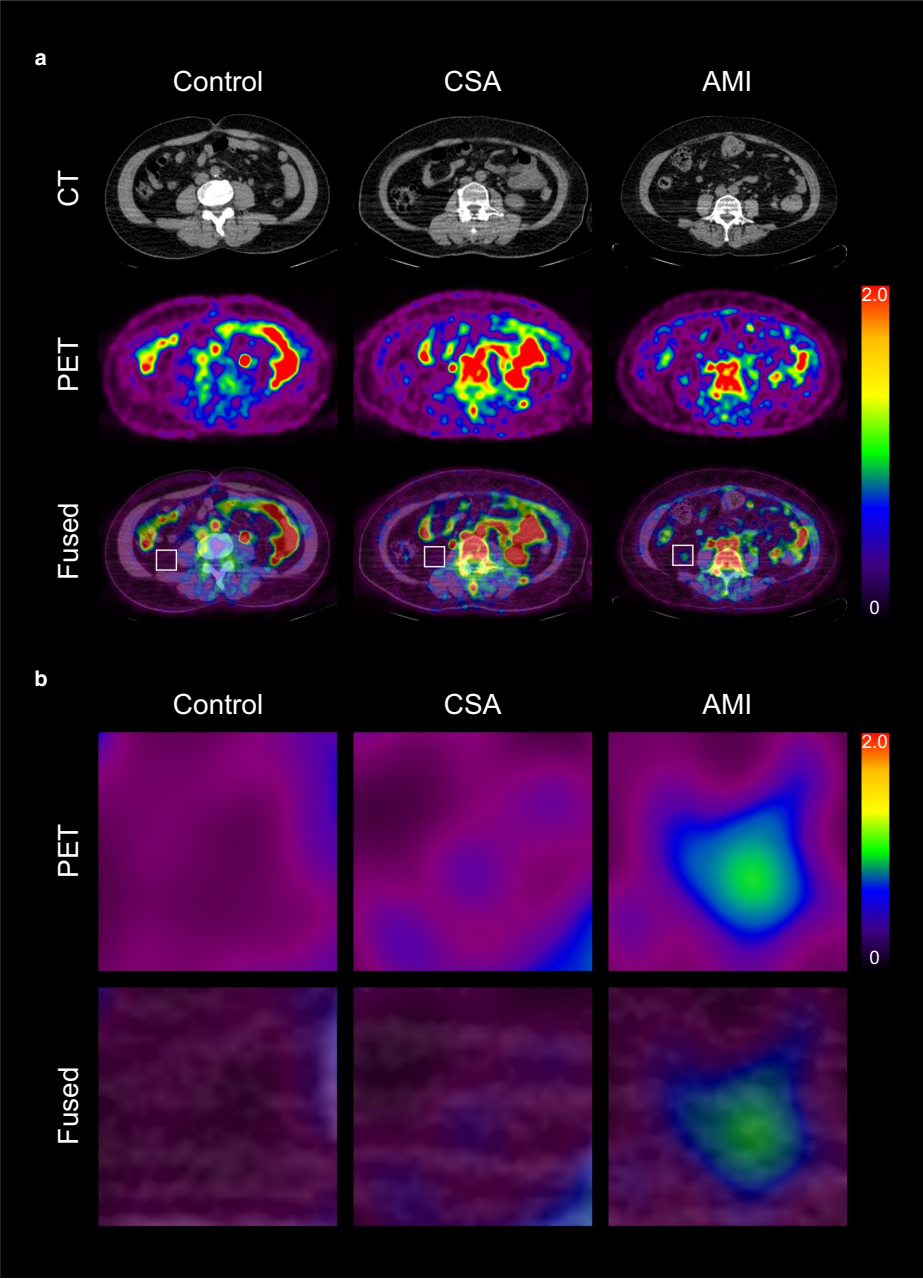
Representative images of visceral adipose tissue (VAT) glucose uptake according to the severity of coronary artery disease (CAD) (**a**), and their corresponding magnified views (**b**). *CSA* chronic stable angina, *AMI* acute myocardial infarction, *CT* computed tomography, *PET* positron emission tomography

**Fig. 2:**
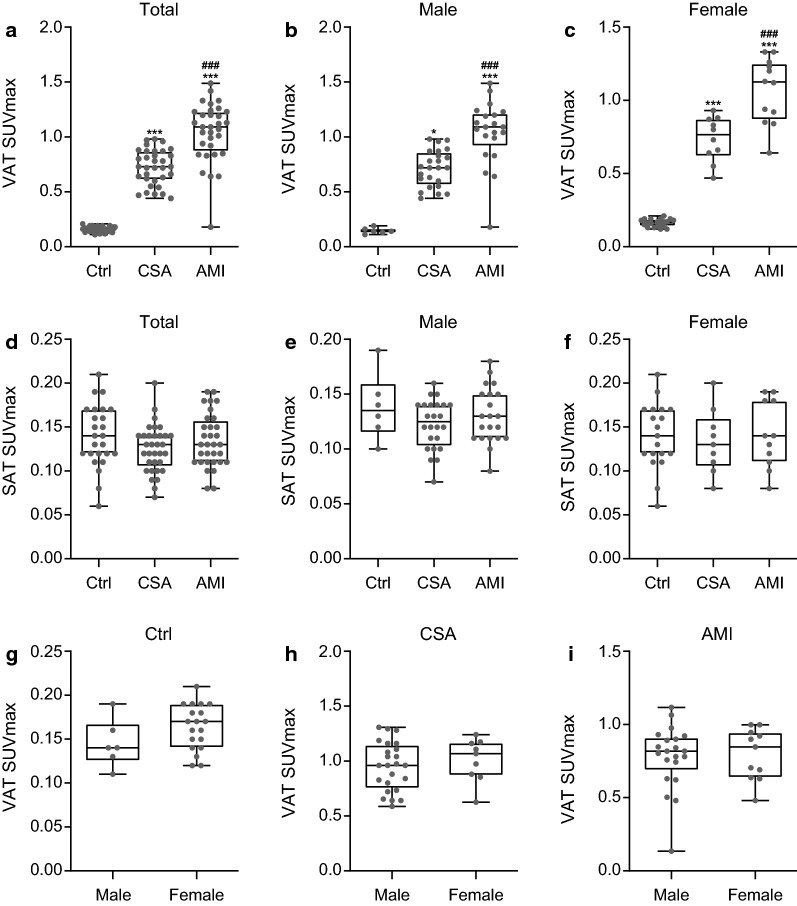
Comparison of VAT SUVmax and SAT SUVmax in total- (**a**, **d**), male- (**b**, **e**), and female participants (**c**, **f**), according to the severity of CAD. Comparison of VAT SUVmax between male- and female participants in Ctrl (**g**), CSA (**h**), and AMI (**i**) group. **a**, **d**; Ctrl, *n* = 25; CSA, *n* = 33; AMI, *n* = 32. Ctrl, control; SUVmax, maximum standardized uptake value; SAT, subcutaneous adipose tissue. *P*-values were determined using Kruskal–Wallis test with post hoc Dunn’s test. ***p < 0.001; vs. Ctrl, ^###^ *p* < 0.001; vs. CSA. **b**, **e**; Ctrl, *n* = 6; CSA, *n* = 24; AMI, *n* = 21. *P*-values were determined using Kruskal–Wallis test with post hoc Dunn’s test. ***p < 0.001; vs. Ctrl, * *p* < 0.05; vs. Ctrl, ^###^ *p* < 0.001; vs. CSA. **c**, **f**; Ctrl, *n* = 19; CSA, *n* = 9; AMI, *n* = 11. *P*-values were determined using one-way analysis of variance (ANOVA) with post hoc Tukey test. ***p < 0.001; vs. Ctrl, ^###^ *p* < 0.001; vs. CSA. **g**; male, *n* = 6; female, *n* = 19. *P*-values were determined using Student’s *t*-test. **h**; male, *n* = 24; female, *n* = 9. *P*-values were determined using Student’s *t*-test. **i**; male, *n* = 21; female, *n* = 11. *P*-values were determined using Mann–Whitney *U* test

We first investigated whether the VAT glucose uptake was increased in CAD group through the comparison of multiple groups. As shown in Fig. 1 and 2a, VAT SUVmax was highest in participants with AMI, intermediate in participants with CSA, and lowest in control group (1 ± 0.3 vs. 0.7 ± 0.2 vs. 0.2 ± 0.03, *p* < 0.001, respectively). AMI group showed significant higher VAT SUVmax than CSA and control groups (*p* < 0.001). Furthermore, VAT SUVmax of CSA was also significantly higher than that of control group (*p* < 0.001). However, there was no significant difference of SAT SUVmax among the three groups (AMI; 0.13 ± 0.03 vs. CSA; 0.13 ± 0.03 vs. Control; 0.14 ± 0.04, Fig. 2d). These results from total participants were not different in both male- and female participants (Fig. 2b, c, e, and f). Furthermore, in subgroup analysis, there was also no significant difference between the VAT SUVmax and SAT SUVmax of male- and female participants in control (0.15 ± 0.03 vs. 0.16 ± 0.2, *p *= 0.14; 0.14 ± 0.03 vs. 0.14 ± 0.04, *p* = 0.98, respectively), CSA (0.71 ± 0.17 vs. 0.76 ± 0.15, *p *= 0.5; 0.12 ± 0.02 vs. 0.13 ± 0.04, *p* = 0.31, respectively), and AMI groups (1.04 ± 0.29 vs. 1.06 ± 0.23, *p *= 0.14; 0.13 ± 0.03 vs. 0.14 ± 0.04, *p* = 0.98, respectively) (Fig. 2g to i).

### Arterial- and systemic inflammation is increased in CAD

Next, we examined whether the arterial- and systemic inflammation was increased in CAD group through the comparison of multiple groups. Both AMI- and CSA group showed significant higher carotid artery TBR than control group (Table 2). Furthermore, carotid artery TBR was significantly increased in AMI group than that in CSA group. Spleen-, BM SUVmax, and hsCRP, well-known surrogate markers for systemic inflammation [16, 18], were significantly greater in CAD group than that in control group. In addition, the participants with AMI exhibited significant higher systemic inflammatory status compared to the participants with CSA, as expected (Table 2).

### Relationship between VAT glucose uptake and arterial- and systemic inflammation

We performed correlation analysis to evaluate the relationship between VAT glucose uptake and arterial- and systemic inflammation. As shown in Table 3, VAT SUVmax showed strong correlation with carotid artery TBR and surrogate markers for systemic inflammation. In contrast, we could not find any correlation between SAT SUVmax and arterial- and systemic inflammation.

**Table 3:** Spearman correlation analysis between metabolic parameters of adipose tissue, systemic-, arterial inflammation parameters, and adipose tissue area

	VAT SUVmax		SAT SUVmax	
	*r*	*p*	*r*	*p*
Carotid artery TBR	0.543	< 0.001*	− 0.064	0.548
Spleen SUVmax	0.674	< 0.001*	− 0.108	0.312
BM SUVmax	0.686	< 0.001*	− 0.177	0.095
hsCRP	0.513	< 0.001*	− 0.073	0.506
VAT area	0.326	< 0.01*	− 0.202	0.061
SAT area	− 0.099	0.364	− 0.141	0.192

Data were correlation coefficients from correlation analysis

*VAT* visceral adipose tissue, *SAT* subcutaneous adipose tissue, *SUVmax* maximum standardized uptake value, *TBR* target-to-background ratio, *BM* bone marrow, *hsCRP* high-sensitivity C-reactive protein

* Statistically significant difference

Next, we performed uni- and multivariate linear regression analyses to support the correlation analysis. Univariate analysis showed that carotid artery TBR was significantly associated with dyslipidemia, hsCRP, spleen SUVmax, BM SUVmax, and VAT SUVmax (Table 4). In further multiple regression analyses, spleen SUVmax and VAT SUVmax were independently associated with carotid arterial TBR (*R*^2^ = 0.431) (Table 4).

**Table 4:** Univariate- and multivariate analyses for carotid artery TBR values

	Univariate		Multivariate	
Variable	Coefficients (95% CI)	*p*	Coefficients (95% CI)	*p*
Age (Continuous)	− 0.004 (− 0.017 to 0.009)	0.553		
Sex (Female vs Male)	0.253 (− 0.029 to 0.535)	0.078		
BMI (Continuous)	− 0.01 (− 0.053 to 0.033)	0.657		
WC (Continuous)	− 0.006 (− 0.016 to 0.005)	0.318		
HTN (Negative vs Positive)	0.026 (− 0.272 to 0.324)	0.862		
DM (Negative vs Positive)	0.28 (− 0.026 to 0.585)	0.072		
Dyslipidemia (Negative vs Positive)	0.33 (0.044 to 0.615)	0.024*	0.026 (− 0.231 to 0.283)	0.839
Current smokers (None vs Yes)	0.1 (− 0.215 to 0.416)	0.53		
Statin use (None vs Yes)	0.309 (− 0.031 to 0.648)	0.074		
Hypertension medication (None vs Yes)	− 0.065 (− 0.393 to 0.263)	0.694		
DM medication (None vs Yes)	0.242 (− 0.08 to 0.563)	0.139		
hsCRP (Continuous)	0.01 (0.003 to 0.018)	0.008*	0.004 (− 0.003 to 0.01)	0.278
Spleen SUVmax (Continuous)	0.744 (0.526 to 0.962)	< 0.001*	0.401 (0.094 to 0.707)	0.011*
BM SUVmax (Continuous)	0.438 (0.271 to 0.606)	< 0.001*	− 0.003 (− 0.252 to 0.245)	0.98
VAT SUVmax (Continuous)	1.003 (0.715– 1.291)	< 0.001*	0.611 (0.14 to 1.083)	0.012*
SAT SUVmax (Continuous)	− 1.417 (− 6.04 to 3.206)	0.544		
VAT area (Continuous)	− 0.000044 (− 0.002 to 0.001)	0.954		

*BMI* body mass index, *WC* waist circumference, *HTN* hypertension, *DM* diabetes mellitus, *hsCRP* high-sensitivity C-reactive protein, *SUVmax* maximum standardized uptake value, *BM* bone marrow, *VAT* visceral adipose tissue, *SAT* subcutaneous adipose tissue, *CI* confidence interval

* Statistically significant difference

### VAT glucose uptake is independently associated with AMI

**Fig. 3:**
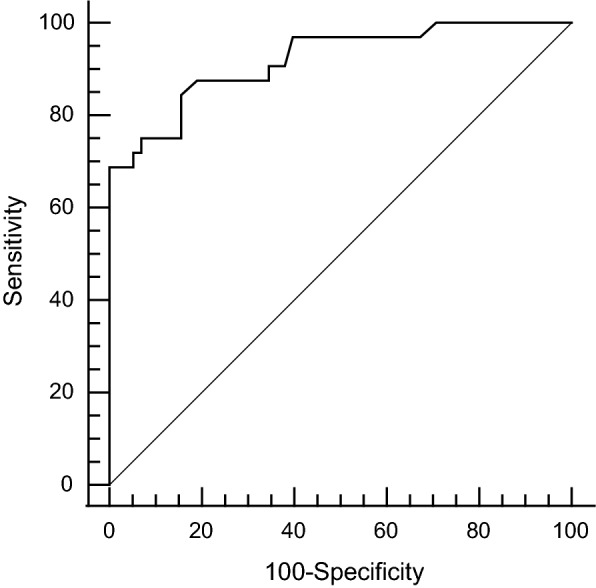
Receiver-operating characteristics (ROC) curve analysis to identify AMI. The Optimal cut-off value was determined by choosing the point corresponding to maximum Youden index (sensitivity-[1-specificity]) on the ROC curve

We performed ROC curve analysis to define the optimal cut-off VAT SUVmax to identify AMI. According to the ROC curve analysis, the optimal cut-off VAT SUVmax to identify AMI was 0.83 with a sensitivity of 84.4% and a specificity of 84.5% (Fig. 3). Area under the curve (AUC) was 0.92 (95% confidence interval 0.84–0.97; standard error 0.03; *p* < 0.001).

Next, using the optimal cut-off VAT SUVmax, we performed multivariate logistic regression analyses to evaluate the association between VAT SUVmax and AMI. As shown in Table 5, multivariate analyses showed that dyslipidemia and VAT SUVmax were significantly associated with AMI and among the included variable, VAT SUVmax showed the highest odds ratio for AMI.

**Table 5:** Univariate- and multivariate analyses for acute myocardial infarction

Variable	Univariate		Multivariate	
	OR (95% CI)	*p*	OR (95% CI)	*p*
Age (Continuous)	0.978 (0.939–1.019)	0.978		
Sex (Female vs Male)	1.782 (0.73–4.352)	0.205		
BMI (Continuous)	0.974 (0.853–1.111)	0.692		
WC (Continuous)	0.976 (0.941–1.012)	0.191		
HTN (Negative vs Positive)	0.541 (0.707–4.175)	0.232		
DM (Negative vs Positive)	1.87 (0.745–4.696)	0.183		
Dyslipidemia (Negative vs Positive)	3.085 (1.253–7.598)	0.014*	3.971 (1.112–14.174)	0.034*
Current smokers (None vs Yes)	1.733 (0.688–4.369)	0.244		
Statin use (None vs Yes)	1.601 (0.581–4.413)	0.363		
Hypertension medication (None vs Yes)	0.543 (0.189–1.557)	0.256		
DM medication (None vs Yes)	1.364 (0.521–3.567)	0.527		
VAT SUVmax (≤ 0.83 vs > 0.83)	29.4 (8.945–96.628)	< 0.001*	31.757 (8.765–115.055)	< 0.001*
VAT area (Continuous)	1 (0.995–1.004)	0.915		

BMI, body mass index; WC, waist circumference; HTN, hypertension; DM, diabetes mellitus; SUVmax, maximum standardized uptake value; VAT, visceral adipose tissue; OR, odds ratio; and CI, confidence interval

* Statistically significant difference

### Relationship between VAT SUVmax and VAT area

We performed correlation analysis to evaluate the relationship between metabolic parameters of adipose tissue and adipose tissue area. As shown in Table 3, VAT area showed significant positive correlation with VAT SUVmax, whereas it showed no significant correlation with SAT SUVmax. Regarding SAT area, it showed no significant correlation with VAT SUVmax and SAT SUVmax. Next, we performed uni- and multivariate linear regression analyses for VAT SUVmax. Univariate analysis showed that VAT SUVmax was significantly associated with age, sex, hypertension, diabetes mellitus, dyslipidemia, current smoking habit, hsCRP, spleen SUVmax, BM SUVmax, and VAT area (Table 6). In further multiple regression analyses, spleen SUVmax and BM SUVmax were independently associated with VAT SUVmax (*R*^2^ = 0.664) (Table 6).

**Table 6:** Univariate- and multivariate analyses for VAT SUVmax

	Univariate		Multivariate	
Variable	Coefficients (95% CI)	*p*	Coefficients (95% CI)	*p*
Age (Continuous)	0.003 (− 0.004 to 0.011)	0.039*	0.002 (− 0.004 to 0.008)	0.486
Sex (Female vs male)	0.236 (0.074 to 0.399)	0.005*	− 0.007(− 0.141 to 0.127)	0.92
BMI (Continuous)	0.02 (− 0.005 to 0.045)	0.113		
WC (Continuous)	0.002 (− 0.004 to 0.009)	0.476		
HTN (Negative vs Positive)	0.256 (0.091 to 0.421)	0.003*	0.036 (− 0.094 to 0.165)	0.586
DM (Negative vs Positive)	0.206 (0.03 to 0.382)	0.023*	0.075 (− 0.06 to 0.21)	0.088
Dyslipidemia (Negative vs Positive)	0.273 (0.112 to 0.434)	0.001*	− 0.011 (− 0.14 to 0.119)	0.867
Current smokers (None vs Yes)	0.218 (0.045 to 0.392)	0.014*	0.132 (− 0.015 to 0.279)	0.077
hsCRP (Continuous)	0.006 (0.001 to 0.01)	0.013*	0 (− 0.003 to 0.004)	0.774
Spleen SUVmax (Continuous)	0.519 (0.404 to 0.634)	< 0.001*	0.302 (0.16 to 0.443)	< 0.001*
BM SUVmax (Continuous)	0.404 (0.329 to 0.478)	< 0.001*	0.205 (0.086 to 0.325)	0.001*
VAT area (Continuous)	0.001 (0.000 to 0.002)	0.002*	0.001 (0 to 0.001)	0.12
SAT area (Continuous)	0 (− 0.001 to 0)	0.308		

*BMI* body mass index, *WC* waist circumference, *HTN* hypertension, *DM* diabetes mellitus, *hsCRP* high-sensitivity C-reactive protein, *SUVmax* maximum standardized uptake value, *BM* bone marrow, *VAT* visceral adipose tissue, *SAT* subcutaneous adipose tissue, *CI* confidence interval

* Statistically significant difference

## Discussion

To the best of our knowledge, this is the first study to assess the relationship between VAT glucose uptake and the severity of CAD in human participants by using ^18^F-FDG PET/CT. In the present study, we demonstrated that VAT glucose uptake defined as VAT SUVmax was highest in participants with AMI, intermediate in participants with CSA, and lowest in control participants. VAT SUVmax was significantly correlated with arterial inflammation and surrogate markers for systemic inflammation. Furthermore, it was independently associated with carotid artery TBR and AMI even after consideration of other risk factors.

Several preclinical studies have been suggested that CAD evokes increased circulating monocytes and increased accumulation of macrophages in remote atherosclerotic lesions, which could lead to another CAD event such as recurrent MI [19–21]. This finding is robustly supported by various clinical studies by using ^18^F-FDG PET/CT that both systemic inflammation markers and carotid artery inflammation are increased in CAD patients according to the severity of CAD and associated with each other [16, 18, 22], which are also consistent with our results.

Inflamed and dysfunctional VAT is central to the development of systemic- and remote arterial inflammation through the secretion of multiple pro-inflammatory cytokines such as TNF- α, IL-6, leptin, and resistin into systemic circulation [4–6]. Thus, next we investigated the relationship between VAT glucose uptake and systemic- and remote arterial inflammation. In this study, we found that VAT glucose uptake defined as VAT SUVmax was significantly associated with the carotid artery inflammation, as well as with the systemic inflammation. More recently, we also find that VAT SUVmax is correlated with systemic inflammation and the number of metabolic syndrome component [13]. Therefore, collectively, these findings suggest that VAT SUVmax can reflect the inflammatory burden of VAT which interacts with remote organ such as vasculature that eventually leads to escalation of CVD risk.

Although the classical anthropometric measurements such as BMI or WC are easily obtainable and have been used to evaluate the obesity-related risk of CAD [23], recent studies have reported that these parameters are not sufficient to evaluate the risk of CAD and have suggested that volumetric measurement of fat depots such as VAT area by CT or magnetic resonance imaging (MRI) could be used to assess the risk of CAD [24, 25]. However, volumetric measurement of VAT as a structure-based approach could not fully capture the dysfunctional metabolic activity of VAT, a key underlying mechanism of obesity-driven CAD risk [26, 27]. Consistent with previous findings, in the present study, we observed that BMI, WC, and VAT area were not associated with carotid artery TBR, which is known to associate with the risk of developing future CAD [18, 28], in contrast to VAT SUVmax. This finding suggests the possibility that the glucose uptake of VAT may be strong predictor of VAT-associated CAD than the volume of VAT. Therefore, based on our study, VAT SUVmax evaluated by ^18^F-FDG PET/CT could be more suitable to assess the obesity-driven risk of CAD than other conventional anthropometric measurements.

In the present study, we found that there was no significant difference of VAT SUVmax between male and female in all CAD and control groups, which was consistent with previous study [26]. Thus, VAT SUVmax seems to be less affected by gender status. However, in the present study, the number of female in the control group was far more than in the CSA or the AMI group which could be a major bias. Thus, considering the different modulation of VAT biology and metabolism by sex differences [29], further large study is warranted to explore the underlying detailed mechanism between VAT inflammation and sex differences.

A growing body of evidence suggests the therapeutic potential of targeting inflamed VAT to treat or prevent CVD [30]. Furthermore, recently, several healthcare professional organizations recommend lifestyle modification, pharmacological, and surgical interventions for visceral obesity management and are becoming increasingly focused on the evaluation of VAT inflammation for risk stratification and treatment response assessment to maximize clinical benefit [31, 32]. Although VAT biopsy can be considered as a gold standard to evaluate VAT inflammation, implementation of tissue biopsy into clinical practice remains a challenge due to the cumbersome biopsy procedure. Interestingly, in previous study, the patients with metabolic syndrome taking anti-diabetic, anti-hypertensive, or lipid lowering drugs which are known to have anti-inflammatory effect show reduced VAT SUVmax [13]. Thus, instead of biopsy, it would be feasible to use VAT SUVmax as a surrogate marker of VAT inflammation in clinical practice.

Recently, several randomized clinical trials have demonstrated that both novel drugs glucagon-like-peptide-1 (GLP-1) receptor agonist and sodium glucose cotransporter 2 inhibitors (SGLT2i) can reduce visceral adiposity thereby attenuating CVD risks [33–35]. Considering that the inflamed VAT is a causal mechanism which contributes to the development of CVD, it is conceivable that VAT SUVmax could be employed as a potential surrogate marker of VAT inflammation, thereby evaluating therapeutic effect of both GLP-1 receptor agonist and SGLT2i targeting inflamed VAT and its related CVD risk.

This study has several limitations. First, the present study was conducted at single center with cross-sectional design and small sample size, which may induce selection bias. Further studies with larger populations are necessary to confirm our findings. Second, unlike AMI and CSA, we were unable to perform the coronary angiography in the control group to assess the extent of coronary atherosclerosis. Third, although ^18^F-FDG PET/CT has been widely used for the assessment of VAT glucose uptake [10–13], we could not perform VAT biopsy to obtain tissue samples from VAT, which might support our findings. Finally, we could not control all the possible factors that could influence ^18^F-FDG uptake including plasma insulin and glucose levels, nor the image acquisition time after ^18^F-FDG injection.

## Conclusion

We observed that VAT glucose uptake, defined as VAT SUVmax and assessed by ^18^F-FDG PET/CT, was associated with the severity of CAD and synchronized with the arterial inflammation which may lead to the future CVD events. These findings provide additional evidence for VAT SUVmax as an imaging surrogate marker for visceral obesity-driven CVD risk. Furthermore, our results offer insights into exploring the interplay between VAT glucose uptake and the severity of CAD.

## Data Availability

The datasets used and/or analyzed during the current study are available from the corresponding author on reasonable request.

## Abbreviations

AMI: Acute myocardial infarction
BM: Bone-marrow
BMI: Body mass index
CAD: Coronary artery disease
CSA: Chronic stable angina
CVD: Cardiovascular disease
hsCRP: High-sensitivity C-reactive protein
SAT: Subcutaneous adipose tissue
SUV: Standardized uptake value
TBR: Target-to-background ratio
VAT: Visceral adipose tissue
^18^F-FDG PET/CT: ^18^F-fluorodeoxyglucose positron emission tomography
